# How does this make you feel? A comparison of four affect induction procedures

**DOI:** 10.3389/fpsyg.2014.00689

**Published:** 2014-07-08

**Authors:** Xuan Zhang, Hui W. Yu, Lisa F. Barrett

**Affiliations:** ^1^Department of Psychology, Boston College, Chestnut HillMA, USA; ^2^Department of Psychology, Northeastern UniversityBoston, MA, USA; ^3^Department of Psychiatry and The Martinos Center for Biomedial Imaging, Massachusetts General Hospital/Harvard Medical SchoolBoston, MA, USA

**Keywords:** affect induction, procedure, self-report, comparison, efficacy

## Abstract

Affect is a fundamental aspect of the human mind. An increasing number of experiments attempt to examine the influence of affect on other psychological phenomena. To accomplish this research, it is necessary to experimentally modify participants' affective states. In the present experiment, we compared the efficacy of four commonly used affect induction procedures. Participants (38 healthy undergraduate students: 18 males) were randomly assigned to either a pleasant or an unpleasant affect induction group, and then underwent four different affect induction procedures: (1) recall of an affectively salient event accompanied by affectively congruent music, (2) script-driven guided imagery, (3) viewing images while listening to affectively congruent music, and (4) posing affective facial actions, body postures, and vocal expressions. All four affect induction methods were successful in inducing both pleasant and unpleasant affective states. The viewing image with music and recall with music procedures were most effective in enhancing positive affect, whereas the viewing image with music procedure was most effective in enhancing negative affect. Implications for the scientific study of affect are discussed.

## INTRODUCTION

For centuries, philosophers have believed that every moment of waking life is to some degree pleasant or unpleasant with some degree of arousal, so that affect is a basic ingredient of mental life. The term “affect” refers to a neuropsychologically basic state that can be described as hedonic (pleasant or unpleasant) with some degree of arousal (from sleepy to activated; for a review, see Lang et al., 1999; Russell and Barrett, 1999). Consistent with philosophers’ musings, research over the past several decades has illustrated that affect is a central feature in emotion (Diener et al., 1999; Russell, 2003; Barrett, 2006a,b), and exerts influence on many psychological phenomena, including vision (for a review, see Barrett and Bar, 2009), attitudes (e.g., Ito and Cacioppo, 2001), personality (e.g., Revelle, 1995; Yik et al., 2002), stereotyping, and prejudice (e.g., Forgas and Fiedler, 1996),verbal communication and negotiation strategies (e.g., Forgas, 1998, 1999), judgment and decision-making (e.g., Forgas, 1995; Greene and Haidt, 2002), predicting the future (e.g., Gilbert and Ebert, 2002), work motivation (e.g., Seo et al., 2004), psychopathology (e.g., Davidson et al., 2002), health (Gallo et al., 2005), and well-being (e.g., Davidson, 2004). Affect provides a common metric (or what neuroeconomists call a “common currency”) for comparing qualitatively different events (Cabanac, 2002), and can serve as the basis for moral judgments of right and wrong (Haidt, 2001). Therefore, in order to experimentally and systematically study the influence of affect and examine its consequences for other psychological phenomena, it is crucial to not only succesfully but also most effectively induce brief/transit changes in a perceiver’s affective state. A variety of affect induction procedures have been designed and tested in prior research, with varying degrees of success. In this paper, with an aim to find the most effective affect induction procedure, we choose four most commonly used affect induction procedures from the previous literature and directly compare their efficacy to change a person’s self-reported affective state. 

### TYPES OF AFFECT INDUCTION

One type of affect induction procedure often used in the experimental literature relies on participants remembering the past or imagining the future to cultivate the desired affective state. The Music and Contemplation in Idiographic context technique (MCI; Eich et al., 2007) combines emotionally evocative music with self-generated imagery. In some studies, participants imagine hypothetical or real situations while listening to music to elicit the desired affective state. MCI has successfully induced affect to study memory (e.g., Eich et al., 1994) and the psychological construction of emotion (Lindquist and Barrett, 2008). In another variation, participants recall and relive memories of affectively significant past events to generate a change in their affective state (Goodwin and Williams, 1982). Recall-based affect induction has successfully induced affect to study its impact on visual attention (Jefferies et al., 2008), social judgment (Bodenhausen et al., 1994), and persuasion (Brinol et al., 2007).

A second type of affect induction that is common in the laboratory relies on participants generating their own mental imagery under detailed guidelines given by the researcher. In this induction method, participants read vignettes to guide their imagination, such as “You buy a lottery ticket and you win $100.00 instantly,” after listening to a piece of music for one minute before the imagination and also with the music continuing in the background during the imagination (Mayer et al., 1990, 1995). Alternatively, participants listen to and immerse themselves in the affectively salient scenarios instead of reading them. This guided imagery has been used to induce affect to study expectations and self-evaluations (Wright and Mischel, 1982) and standards for performance (Wood et al., 2009). Guided imagery combined with music was also used in the study of the influence of affect on cognitive tasks including memory and facial emotion recognition (Chepenik et al., 2007). 

Another type of affect induction involves viewing affective stimuli, such as photographs or films. A comedic video or a photograph of a smiling baby usually causes people to feel elated. On the contrary, an ominous scene from a horror movie often causes people to feel scared. Photographs from the International Affective Picture System (IAPS; Lang et al., 1999) are widely used to study the influence of affect on attention (Bradley et al., 2003), pain tolerance (Meagher et al., 2001), physiology (Smith et al., 2005), and visual attention (Pereira et al., 2006). Image-based induction procedures have been combined with music in various studies to examine the influence of affective changes on smoking behaviors (Conklin and Perkins, 2005; Perkins et al., 2008) and emotion perception (Baumgartner et al., 2006a). Films have also been used as an affect induction technique in studies assessing mind wandering (Smallwood et al., 2009), emotional coherence (Mauss et al., 2005), and empathy (Davis et al., 1987). A combination of film and music has also been used to study the influence of affect on social judgments and categorization (Halberstadt and Niedenthal, 1997; Innes-Ker and Niedenthal, 2002).

The fourth type of affect induction involves the generation of affectively relevant behaviors with the expectation that changes in affective feeling will follow. Previous studies have shown that when people are asked to assume certain posed facial actions, body postures, and vocal expressions, they tend to report affective experiences that match these emotional behaviors (Duclos et al., 1989; Siegman and Boyle, 1993; Hatfield and Hsee, 1995; Flack et al., 1999; Reisenzein and Studtmann, 2007). Facial expressions have been manipulated to study their effect on psychosomatic states memory (Laird et al., 1989; Schnall and Laird, 2003). Vocal behaviors have been manipulated to study their effect on anger (Siegman et al., 1990), fear and anxiety, and sadness and depression (Siegman and Boyle, 1993). Manipulation of facial expressions, postures, and tone of voice have also been used to study emotional experience (Duclos et al., 1989; Hatfield and Hsee, 1995; Flack et al., 1999; Duclos and Laird, 2001).

### AFFECT INDUCTION METHOD COMPARISON

Given the various procedures available to manipulate a person’s affective state, it has been difficult for researchers to know which technique yields the best results. A small number of early attempts have compared pairs of affect induction procedures, and found autobiographical recollection (Brewer et al., 1980) and music (Clark, 1983) to be superior to the Velten technique, where participants read a series of pleasant or unpleasant self-referential statements (e.g., “I’ve doubted that I’m a worthwhile person”). Attempts to review and compare affect induction procedures started about two decades ago: Westermann et al. (1996) conducted a meta-analysis reporting that affect-inducing materials such as viewing films or presenting stories were most potent for inducing changes in affect, and this finding was also supported by a literature review (Gerrards-Hesse et al., 1994).

Recently, there has been a growing interest in examining the efficacy of different affect induction procedures. A series of studies compared the effects of picture, music, and pictures–music combination on emotional experience induction (Baumgartner et al., 2006a,b). By employing psychometrical, physiological, and neuroimaging measurements of affective states, Baumgartner et al. (2006a,b) found that the combination of music and pictures was the most effective among the three methods. Focusing on *anger* induction, Lobbestael et al. (2008) compared four methods—film, stress interview, punishment, and harassment— and found that although all four produced comparable levels of self-reported anger, two methods that included personal contact (harassment and interview) induced more significant physiological reactivity (Lobbestael et al., 2008). A recent study compared the efficacy of two induction methods, autobiographical recall and a combined procedure of music and guided imagery, finding greater efficiency of the autobiographical recall than of the combined procedure (Jallais and Gilet, 2010). More recently, Vuoskoski and Eerola (2012) used indirect measures (word recall task and judgment task) to compare the *sadness* induced by listening to unfamiliar sad or neutral music, or to self-selected sad music, or recalling a sad autobiographical event. Results indicated that the effects of sad music on memory and judgment depend on the music’s relevance to the listener and how empathy the listener is.

A recent meta-analysis on *discrete emotion elicitation methods* compared their efficacy in inducing four specific emotions (happiness, sadness, anger, and anxiety; Lench et al., 2011). By examining effect sizes from comparisons among discrete emotions for cognitive, judgment, experiential, behavioral, and physiological outcomes and moderators of these effects, the meta-analysis gave us a valuable review of the efficacy of discrete emotion elicitation methods. However, given a further look at the effect sizes they gathered, although participants reported differences in experience across different emotion inductions, they do not show consistent and specific differences in physiological responding and behavior (Lindquist et al., 2013). Moreover, this meta-analysis excluded the articles that did not examine the four targeting discrete emotions, especially those dealt with *general positive and negative affect or mood* (Lench et al., 2011, p. 839). Therefore, a review and comparison of *affect induction methods*, which aim at inducing more general positive versus negative affective states, are in need for the scientific studies of the influence of the more basic affective states rather than specific emotions.

Taken together, these experimental comparisons and meta-analysis attempts gradually add to our knowledge of different affect/emotion induction procedures’ efficacy. However, most studies to date either only focused on a couple of affect induction methods (Brewer et al., 1980; Clark, 1983; Baumgartner et al., 2006a; Jallais and Gilet, 2010) or just targeted one aspect of affective experience (e.g., anger in Lobbestael et al., 2008; sadness in Vuoskoski and Eerola, 2012). To provide a better-rounded picture of major affect induction techniques, a more extensive and inclusive experimental comparison is needed to guide method selection in terms of induction efficacy.

### THE PRESENT STUDY

The current study aimed to systematically compare the efficacy of four affect induction procedures in changing subjective ratings of affective experience: (1) recall with music, (2) guided imagery, (3) viewing images with music, and (4) embodying affective behaviors. To make the arousal level comparable between pleasant and unpleasant condition, we selected angry/anxiety eliciting materials (negative valence high arousal) instead of sadness eliciting materials (negative valence low arousal) to induce unpleasant affective state, and happiness eliciting materials (positive valence high arousal) to induce pleasant affective state. Participants went through either four positive affect inductions or four negative affect inductions, with neutral inductions interspersed in between affect inductions to restore neutral states. Participants’ affective experiences were measured both before and after each induction by two sequential scales representing the hedonic valence and arousal properties of affect (Russell et al., 1989).

## MATERIALS AND METHODS

### PARTICIPANTS

Thirty-eight undergraduate students at Boston College (18 males and 20 females) participated in this experiment. Ages ranged from 18 to 25 (*M* = 19.58, SD = 1.48) and all in healthy condition as required in recruiting procedure. All participants were consented before and debriefed after experiment with IRB-approved forms and compensated with either $15 or 1.5 research credits. One participant’s pre-induction valence rating in the image with music block was outside of the normal range (three standard deviations) and therefore was removed. Altogether, data for 19 participants in the pleasant affect group and 18 participants in the unpleasant affect group were analyzed.

### PROCEDURE

Prior to participating, participants were randomly assigned to one of two affect conditions (pleasant or unpleasant affect group). Upon entering the lab, participants gave informed consent to participate. Next participants were escorted into a study room, where they were left alone to complete each affect induction procedure. We communicated with the participants through a two-way audio system and monitored participant compliance through a closed-circuit SONY video camera mounted on the study room ceiling. Participants were told that the purpose of the study was to assess different methods for changing a person’s feeling state.

For a given experimental session participants received four blocks of inductions (each of the four induction methods). Participants received two sections of inductions in each block. The first section was an affective induction to induce the desired affective state (e.g., pleasant), followed by a second section of neutral induction to restore their affective state back to neutral. 

To provide an example of a typical procedure flow, each participant went through eight trials of induction procedures: Procedure 1 (positive or negative induction) → Procedure 1 (neutral induction) → Procedure 2 (positive or negative induction) → Procedure 2 (neutral induction)→ Procedure 3 (positive or negative induction) → Procedure 3 (neutral induction) → Procedure 4 (positive or negative induction) → Procedure 4 (neutral induction). Block order was randomized for the four procedures. 

The neutral induction was included in each block for several reasons: (1) to serve as a control condition for each affect induction procedure, (2) to restore participants’ affective state back to neutral before the next affect induction procedure was administered to avoid ceiling effects, (3) to provide a pre-induction rating of affect for the next affect induction procedure, and (4) to ensure that participants did not leave the lab in an altered affective state. The neutral induction was designed to match the affective inductions as closely as possible, so identical instructions were given to pleasant, unpleasant, and neutral inductions to account for demand characteristics.

### AFFECT RATINGS

Participants rated their affective state on two 9-point scales representing the hedonic valence and arousal properties of affect (Russell et al., 1989). Participants were asked “How pleasant are you currently feeling?” and “How aroused are you currently feeling?" The 9-point scales were anchored at 1 = extremely unpleasant or low arousal/sleepy, 5 = neutral, 9 = extremely pleasant or high arousal/activated).

### AFFECT INDUCTION PROCEDURES AND MATERIALS

#### Recall with music

Participants recalled the angriest or funniest experiences they could think of while listening to an affect-congruent musical piece intended to induce either an unpleasant or a pleasant state, respectively. Autobiographical narrative sheets were provided for participants to write out an angry (unpleasant), funny (pleasant), or non-evocative (neutral) event. Three musical excerpts from a previous study that successfully manipulated participants’ affect (Conklin and Perkins, 2005) were selected to combine with Recall affect induction procedure. In particular, we chose “The Arrival of The Queen of Sheba”^1^ to induce pleasant affect, “Battle on the Ice”^2^ to induce unpleasant affect, and “Wind on Water”^3^ to induce neutral affect. The music was emitted through two ceiling mounted speakers with stereo sound capability. A small microphone was placed half of a meter away from the participants to record their spoken narratives. Audacity (Dominic Mazzoni, 1.1.2) was used to record and to replay participants’ narratives from the control room computer. Participants were told that the music would help them re-experience the events without explicit instruction on the affective content of the music. Participants were given 10 min to think and write out the event in as much detail as possible. After the participants finished writing the narratives, they read each one aloud while the experimenter recorded them. Finally, participants listened to the playback of their recorded narratives and were asked to relive the experience as vividly as possible. Immediately after the playback, participants provided their affect ratings on valence and arousal.

#### Guided imagery

We developed nomothetic scripts describing experiences that would induce affective changes. In contrast to idiographic scripts, nomothetic scripts described more universal experiences to which all individuals could relate. The pleasant script described a birthday surprise; the unpleasant script described someone cutting in line; and the neutral script described walking down a street. Scripts were read in second person present tense and guided participants’ mental imagery. One of the authors, Lisa F. Barrett, recorded the scripts in a studio at Boston College for playback. We incorporated realistic sound effects such as door opening, footsteps, and the sounds of other people to stimulate vivid mental imagery. The recording times for the three scripts were: 1.37 s for the pleasant script, 1.33 s for the unpleasant script, and 1.05 s for the neutral script. Participants were instructed to listen carefully to scenarios and picture the event in their mind as vividly as possible. Participants were also told to allow their senses to respond to the situation being described and experience the feelings associated with the events. While listening to the scripts, participants closed their eyes. Participants imagined the scenario for 20 s and rated their feelings afterward.

#### Visual images with music

Three videos displaying affectively evocative images combined with music were developed using Windows Movie Maker (Microsoft Corporation, Windows movie maker 2.1). Each video was three minutes long. Images appeared on screen for 5 s each as music played in the background. The images were either selected from the IAPS (Lang et al., 1999) or gathered online. For the set of online images, a pilot study was conducted to collect normative valence and arousal ratings on the scale from 1 to 9. The 36 images with the strongest positive rating were selected for the pleasant video (*M*_Valence_ = 7.6, *M*_Arousal_ = 5.2). The 36 images with the strongest negative rating were chosen for the unpleasant video (*M*_Valence_ = 1.5, *M*_Arousal_ = 7.2). The 36 images with the most neutral valance ratings were selected for the neutral video (*M*_Valence_ = 4.9, *M*_Arousal_ = 3.2). Within each video, images were faded in to reduce the abrupt transition from one image to the next. The same pleasant, unpleasant, and neutral musical excerpts described above were incorporated in the videos. The music was faded in at the beginning and faded out at the end of the video and was played through ceiling mounted stereo speakers. Participants viewed the videos in a dimly lit room on a 40-inch LCD widescreen television (Samsung LNT4065F) mounted on the wall and situated one and a half meters away from the armchair. We instructed the participants to focus on each photograph as it appeared. Participants were told to use their imagination to make the images more personal and to allow themselves to be carried into a deeper affective state. The videos were presented using Eprime (Psychology Software Tools, Pittsburgh, PA, USA), which was also used to collect the participants’ self-report ratings before and after the inductions.

#### Embodiment

With this method, participants manipulated their facial actions, postures, and tone-of-voice. Participants were first told to relax all of the muscles of the face and body and then asked to follow the expression and posture manipulation instructions for 10 s. We adopted the instructions for angry facial expression and bodily posture from Duclos et al. (1989), and happy expression and posture from Flack et al. (1999). We developed our own instructions for the neutral condition. We read each sentence of the instructions separately and observed the participants’ compliance through a video camera and only proceeded to the next sentence when the participants correctly performed the behavior. Immediately after participants assumed these expressions and postures, they read short scripts while using speech patterns (pace, rhythm, and pitch) that were congruent with the affective content of the scripts. Participants were asked to sound as happy/angry/neutral as possible. Participants read the first line for practice, during which we gave them feedback.

### DATA ANALYSIS

First, we checked the effectiveness of the neutral induction procedure in bring participants back to their baseline neutral states before each affect induction by comparing the pre-induction valence and arousal ratings for the four induction methods using one-way ANOVAs. Then we conducted two types of manipulation checks to examine if all four affect induction methods were successful in inducing pleasant or unpleasant affective states by a series of *t*-test. The first manipulation check compared valence and arousal ratings right before and after each affect induction (each induction is compared to the neutral induction before it). The second manipulation compared between the affective vs. neutral version of each induction method (e.g., the recall plus music pleasant induction would be compared to the recall plus music neutral induction). Next, we examined the relative efficacy of the four methods with a separate analysis of valence and arousal. For hedonic valence, we calculated valence difference scores (*d*_V_) by subtracting the mean pre-induction value from the post-induction ratings. With these valence difference scores, we conducted two repeated measures ANOVAs, one for pleasant affect group and one for unpleasant affect group. Similarly, to examine the efficacy of the four affect induction procedures in altering arousal levels, we calculated the arousal difference scores (*d*_A_) by subtracting the mean of pre-induction ratings from post-induction ratings and conducted two separate repeated measures ANOVAs for pleasant and unpleasant affect group.

## RESULTS

### BASELINE VALUES

The pre-induction valence and arousal ratings for the four induction methods were compared using one way ANOVAs (comparing participants in the pleasant and unpleasant affect and no significant differences were found, *p*s > 0.05). This indicates that participants of the four induction methods did not differ significantly from each other before the manipulations began. Also, it reveals that neutral induction procedures were effective in bringing the participants back to their baseline affective state after pleasant or unpleasant affect inductions.

### MANIPULATION CHECK

The manipulation checks confirmed that all four affect induction methods were successful in inducing both pleasant and unpleasant affective states. We performed two types of manipulation checks to test whether each affect induction procedure was successful at inducing the desired affective state. In the first method, valence and arousal ratings were compared before and after each affect induction (each induction is compared to the neutral induction before it). The ratings of affective experience before and after each affect induction epoch are presented in **Table 1**. Effect size was computed for post-induction ratings versus pre-induction ratings of each induction method (Cohen, 1988). Please note that we did not use the effect sizes reported in **Table 1** to compare the efficacy between different methods because the pre-induction ratings of each method can be very noisy and influenced by the neutral induction’s effectiveness. Therefore in the second method, valence, or arousal ratings were compared for the affective vs. neutral version of each induction method (e.g., the recall plus music pleasant induction would be compared to the recall plus music neutral induction). By both types of manipulation checks mentioned above, we confirmed that all four affect induction methods were successful in inducing both pleasant and unpleasant affective states (*p*s < 0.01). Furthermore, participants’ arousal levels increased after each affective induction, compared to both pre-induction and neutral induction (*p*s < 0.05).

**Table 1:** Affective ratings and effect sizes before and after each affect induction.

Induction procedure	Valence				Arousal			
	Pre *mean (SE)*	Post *mean (SE)*	Effect size (*d*)	95% CI	Pre *mean (SE)*	Post *mean (SE)*	Effect size (*d*)	95% CI
**Pleasant affect induction**
Recall/music	5.63 (0.29)	7.11 (0.28)	1.29	[0.92, 1.65]	4.05 (0.38)	5.95 (0.37)	1.08	[0.52, 1.64]
Guided imagery	5.58 (0.33)	7.05 (0.37)	1.16	[0.75, 1.56]	4.05 (0.34)	5.95 (0.43)	1.04	[0.47, 1.62]
Image/music	6.37 (0.24)	7.47 (0.29)	1.06	[0.73, 1.39]	4.74 (0.31)	5.68 (0.41)	0.59	[0.09, 1.10]
Embodiment	6.16 (0.32)	6.63 (0.30)	0.44	[0.10, 0.78]	4.21 (0.39)	5.68 (0.35)	0.93	[0.43, 1.43]
**Unpleasant affect induction**
Recall/music	6.17 (0.30)	3.67 (0.29)	1.93	[1.51, 2.35]	3.56 (0.39)	6.22 (0.38)	1.94	[1.49, 2.39]
Guided imagery	5.72 (0.34)	4.11 (0.38)	0.96	[0.41, 1.51]	3.61 (0.35)	6.06 (0.44)	1.77	[1.31, 2.22]
Image/music	6.50 (0.25)	2.39 (0.30)	3.33	[2.93, 3.74]	4.00 (0.32)	6.56 (0.42)	1.69	[1.20, 2.19]
Embodiment	6.22 (0.33)	4.22 (0.31)	1.29	[0.79, 1.80]	4.44 (0.40)	6.61 (0.36)	1.41	[0.91, 1.92]

*Effect sizes were reported as Cohen's d for the difference between post-induction ratings and pre-induction ratings.*

### EFFICACY COMPARISON

Next, we examined the relative efficacy of the four methods to see which methods were most potent in inducing the desired affective state. We separately analyzed the data according to the two basic properties of affect (valence and arousal).

#### Hedonic valence

**FIGURE 1:**
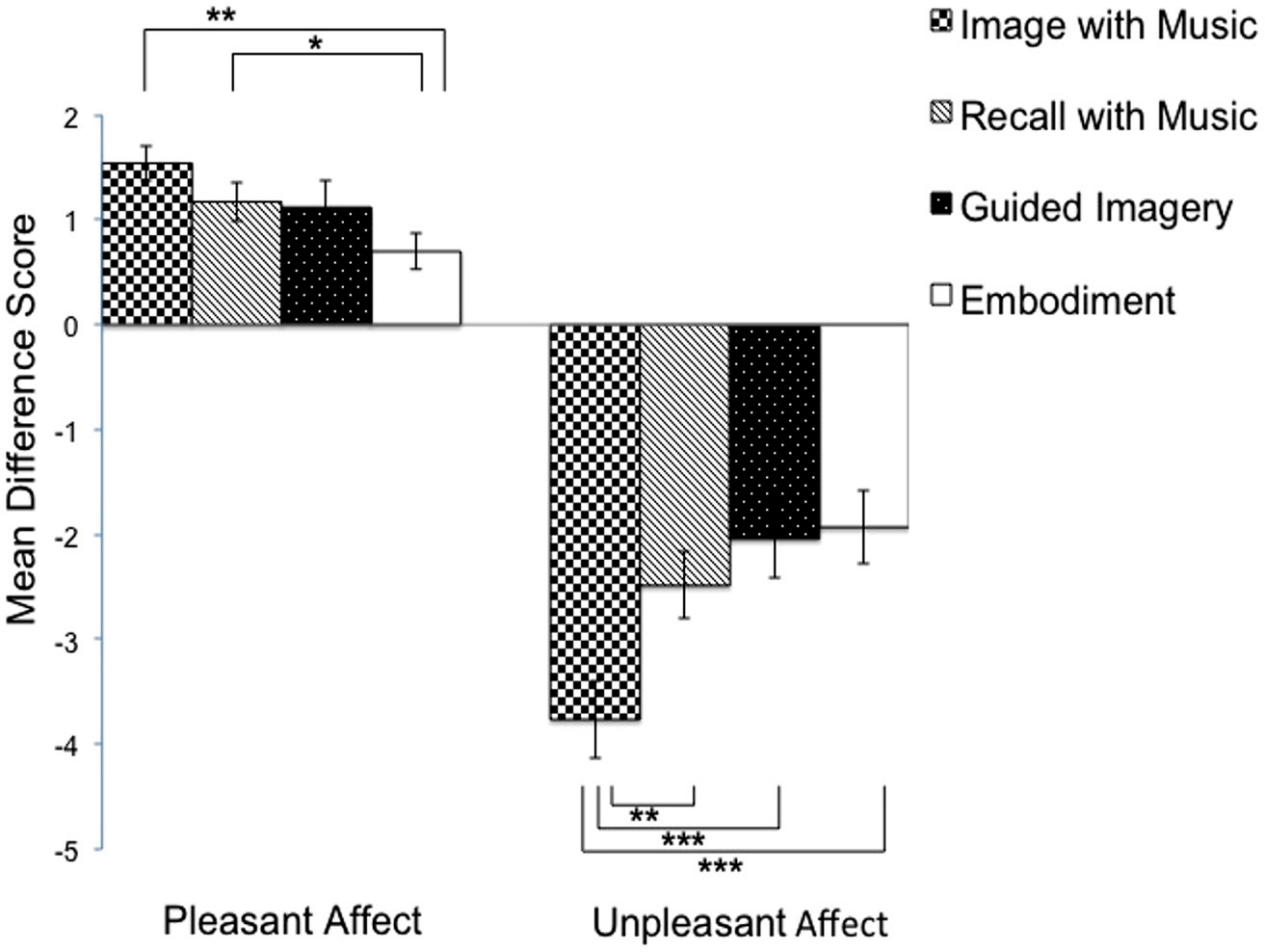
**Mean pre–post difference score of hedonic valence in each affect induction method.** *p < 0.05; **p < 0.01; ***p < 0.001.

To examine the efficacy of four affect induction procedures on hedonic valence, we first calculated the *mean valence rating* across all pre-induction conditions for each group and then calculated valence difference scores (*d*_V_) by subtracting the* pre-induction mean value* from the post-induction ratings. With these valence difference scores, we conducted two repeated measures ANOVAs, one for pleasant affect group and one for unpleasant affect group (see **Figure 1**).

In the pleasant affect group, affect induction procedures were differentially effective, *F* (3,54) = 3.70, *p* = 0.017, η^2^ = 0.17. Contrasts between the four conditions were assessed by means of *post hoc* analyses (Bonferroni tests). Among all pair-wise comparisons, two were significant (**Figure 1**). First, participants felt more intense pleasant affect in the *Image with Music Condition* compared to the *Embodiment Condition*, mean difference = 0.842, CI = (0.232, 1.453), *p* = 0.004, *d* = 0.938 [effect size corrected for dependence between means using Morris and DeShon (2002) Equation 8]. Second participants also reported more intense pleasant affect in the *Recall with Music* compared to the *Embodiment Condition*, mean difference = 0.474, CI = (0.000, 0.947), *p* = 0.05, *d* = 0.684. The other *post hoc* contrasts were not significant.

In the unpleasant affect group, affect induction procedures were also differentially effective, *F*(3,51) = 7.95, *p* < 0.001, η^2^ = 0.32. *Post hoc* contrasts revealed that participants felt more intense unpleasant affect in *Image with Music Condition* as compared to the other three induction method: *Recall with Music Condition*, mean difference = -1.278, CI = (-2.55, -0.006), *p* < 0.05, *d* = 0.711; *Guided Imagery Condition*, mean difference = -1.722, CI = (-3.061, -0.383), *p* < 0.01, *d* = 0.904; and *Embodiment Condition*, mean difference = -1.833, CI = (–2.744, -0.923), *p* < 0.001, *d* = 1.418.

#### Subjective arousal

To examine the efficacy of the four affect induction procedures in altering arousal levels, we calculated the arousal difference scores (*d*_A_) by subtracting the mean of pre-induction ratings from post-induction ratings and conducted two separate repeated measures ANOVAs for pleasant and unpleasant affect group. There was no significant effect of induction methods in either group: for the pleasant group, *F*(3,54) = 0.404, *p* = 0.662, η^2^ = 0.022; and for the unpleasant group, *F*(3,51) = 1.092, *p* = 0.349, η^2^ = 0.06. All affect induction procedures were equally effective in inducing arousal changes, such that participants did not differ in their change of arousal ratings after each pleasant and unpleasant induction across the four affect induction methods.

## DISCUSSION

Our findings confirmed the effectiveness of four most commonly used affect induction procedures in the literature. Asking participants to project him or herself into the past (by recalling a prior event) while listening to music, imagine a fictitious scenario, view photographs while listening to music, and take on affective postures were all successful in inducing both pleasant and unpleasant affective changes. Yet some procedures are more effective than others. Both viewing evocative photographs while listening to music and recalling an affectively salient event while listening to music were most effective in inducing the experience of *pleasant* affect. Viewing evocative photographs while listening to music was the most effective in inducing the experience of *unpleasant* affect. These findings indicate that the combination of presenting an evocative image and music is a powerful way of manipulating the hedonics of an affective state. All four affect induction methods were equally effective in modifying changes in experienced arousal.

Our findings are also in line with Lench et al. (2011) meta-analysis results if we average the effect size comparisons between positive emotion (happiness) and negative emotions (anger, sadness, and anxiety). From Table 4 in Lench et al. (2011) and Table 1 in Lindquist et al. (2013), we can get the average effect size of different induction method for eliciting positive vs. negative emotions: Music (average effect size = 1.23), Pictures (1.08), Film (0.94), Imagine (0.78), Behavior (0.73), Recall (0.62), Velten (0.5), and Real-life manipulations (0.24). With music and picture being the top two in the list, our finding of the combination of music and picture being the most effective in inducing hedonic affect can be well supported and explained. Although we did not directly compare the different conditions of music, picture, and combination of music and picture as in Baumgartner et al.’s (2006a) research, our results no doubted manifested the powerful effect of the combination of music and picture—the most effective procedure in inducing both positive and negative affect.

Interestingly, music seemed to have a magic power when combining with other methods (both image and recall) to induce affect. Was it the music component in the combination methods, or simply the combination of two methods, that made the affect induction more effective? Can music alone produce the effects seen with music plus images or recall? The mechanisms of why and how certain methods are more effective than others will need further investigation. Especially with the recent finding from Vuoskoski and Eerola (2012) where the effects of sad music on memory and judgment depended on the music’s relevance to the listener and how empathy the listener was, as well as our own finding of autobiographical recall being more effective in inducing unpleasant affect, attention should be called to the investigation of the role self-relevancy plays in affect induction methods.

Each induction method has its own costs and benefits that must be considered, of course, when designing an experiment. Recalling prior events while listening to music has the advantage of using personally relevant events. People often reminiscence and daydream to get themselves into certain affective states, which makes recall an easy task for participants to perform. However, there may be a positivity effect where participants are more willing to recall positive rather than negative events and remember positive events in greater detail (Destun and Kuiper, 1999; Kealy et al., 2006). According to Taylor (1991), there is a greater pattern of mobilization-minimization for negative events than for neutral or positive events, in which the powerful and immediate physiological, cognitive, and behavioral responses of the negative events damp down and erase the impact of the events. This response mechanism may explain the differential efficacy of recall induction in cultivating pleasant and unpleasant affect. The disadvantage, of course, is that some people have greater memory capacity and therefore remember events in greater detail than do others and some people have more vivid imagery (Richardson and Taylor, 1982). Furthermore, it is difficult to equate the evocativeness of each person’s memory, and also the age of each memory (which might relate to detail and vividness).

Guided imagery has a well-known benefit of stress reduction and relaxation (Lyles et al., 1982; Holden-Lund, 1988; Kolcaba and Fox, 1999; Walker et al., 1999), which is supported by our finding that guided imagery cultivates a fairly strong pleasant affect. While guided imagery provides more control over the content and age of the imagined scenarios than the recall method, scenarios might not be as personally relevant for each participant, limiting their evocativeness. The disadvantages of guided imagery are similar to the recall method in that some people attend more to the details described in the scenarios than do others, and some have more vivid imagery.

Viewing visual images while listening to music does not rely on participants’ memory or imagination, and so avoids the relative pitfalls of mental time travel. Participants are presented with controlled, vivid visual stimulation that looks very similar to real world objects. Presenting images in a more immersive format (e.g., on a 40-inch flat screen TV) while listening to music on a surround sound system enhances the affective impact of the experience. The disadvantage of using standard stimuli (such as photographs and music), however, is that there might be individual differences in preferences and affective potency, which affects the utility of the technique.

Embodiment techniques are easy to use and do not require any equipment. They also lack a cognitive component because they induce affect solely through modifying body state information, which can be a great advantage to studies that involve cognition as the dependent variable. Whereas recall and guided imagery inductions might require more cognitive processing to elicit the desired effect, this might interfere with the cognitive capacity for subsequent tasks. Body states alone might trigger certain thoughts, however, that enhance affective feeling. Furthermore, this form of induction depends heavily on the participants’ sensitivity to their own internal states (Laird, 1984), and there is tremendous variability in such sensitivity (Barrett et al., 2004).

The induction methods here did not exhaust all the possible avenues for manipulating a person’s affective state, of course. In the future, it would be important to compare these more individual induction techniques to high-impact social interaction techniques, such as the Interpersonal Insult (Harmon-Jones and Sigelman, 2001), Success/Failure (Nummenmaa and Niemi, 2004), Trier Social Stress Test (Kirschbaum et al., 1993), and a social-comparison manipulation (Forgas, 1991). While effective, these social interaction techniques are also time consuming and costly to set up in the lab. The present study demonstrates that there are other relatively low cost procedures that effectively change a person’s momentary affective state.

The present study focused on subjective ratings (self-report) as the measurement of affective experience, which is so far the most commonly used to measure affect induction effect. However, self-report has been criticized to be biased by such factors as social desirability or demand characteristics (Orne, 1962). It would be useful for future studies to employ other measurements of affective impact, such as psychophysiological measurements, EEG, or fMRI, to explore the efficacy differences among these methods and their underlying mechanisms. For example, by means of self-report and physiological measures (blood pressure, heart rate, skin conductance level, and skin conductance response), Lobbestael et al. (2008) compared the effects of four ways of inducing anger: film, stressful interview, punishment, and harassment. They found that although all four methods were comparably effective in eliciting self-reported anger, they differed in their extent of influences on physiological measures such that harassment and stress interview produced the largest cardiovascular effects, film condition produced the lowest increase in electrodermal activity, and only harassment caused a significant increase in all physiological measurements. However, these physiological measures do not substitute for self-reported experience, because subjective experience does not reduce to physical measurements (cf. Barrett, 2006b).

Moreover, future research could also employ indirect measurements of affective states (see review by Västfjäll, 2010; e.g., word recall; count time; distance estimation; emotional picture judgment tasks), which are based on the premise that affective states are accompanied by changes in information processing and behavior. Being relatively free from demand characteristics, these indirect measures do not rely on the participants’ conscious interpretations of their own internal processes, and may reveal a different efficacy pattern of different induction methods as compared to the efficacy pattern revealed by direct self-reports from participants. Further along this line, systematic investigation of the duration of affect-induction effects would also be very informative for affective science researchers.

With the purpose to compare different induction method directly, the present study employed a within-subject design. Although such design can maximally reduce individual variances, it may introduce bias in the measure of affect from one condition to the following conditions and also fatigue that may influence the accuracy of measurement. Therefore more experiments should be carried out to examine the affect induction efficacy of different method individually with different groups of participants.

Last but not least, the current study was conducted in a healthy young undergraduate student sample, and to be cautious, the findings are better to be replicated in other groups such as different age groups and clinical samples before generalized to apply in any age-related and disease-related psychological phenomena. For instance, affect induction procedures have been frequently used to study disease vulnerability such as cognitive biases in remitted depressed patients (Wray et al., 2009). However, it is possible that depression patients, comparing to healthy young undergraduates, may have a different response pattern to various affect induction procedures due to the influence of mental illness. Therefore caution should be taken before applying our current findings to other groups.

## CONCLUSION

This simple study indicated that although all four affect induction methods were successful in inducing both pleasant and unpleasant affective states, they differed in their efficacy. When music is combined with image or recall, it was most effective in enhancing self-state pleasant affect, whereas the combined image/music procedure was most effective in enhancing unpleasant affect. Further research comparing different affect induction procedures by means of other measurements than self-reports, their impact duration, and in other age and disease-related groups, is still needed to get a more comprehensive comparison and appropriate application of affect induction methods for psychology research.

## Conflict of Interest Statement

The authors declare that the research was conducted in the absence of any commercial or financial relationships that could be construed as a potential conflict of interest.

## Footnotes

1 Handel, G. (1715). Moll Flanders. Arrival of the Queen of Sheba.

2 Prokofiev, S. (1938). Battle on the Ice.

3 Fripp, R. (1975). Wind on Water. The Essential Fripp & Eno [CD]. New York: Virgin Records. Track 3.
